# Scanning the Effects of Ethyl Methanesulfonate on the Whole Genome of *Lotus japonicus* Using Second-Generation Sequencing Analysis

**DOI:** 10.1534/g3.114.014571

**Published:** 2015-02-06

**Authors:** Nur Fatihah Mohd-Yusoff, Pradeep Ruperao, Nurain Emylia Tomoyoshi, David Edwards, Peter M. Gresshoff, Bandana Biswas, Jacqueline Batley

**Affiliations:** * Centre of Integrative Legume Research, School of Agriculture and Food Science, The University of Queensland, St Lucia, Brisbane QLD 4072, Australia; † Department of Cell and Molecular Biology, Faculty of Biotechnology and Biomolecular Sciences, Universiti Putra Malaysia, 43400, Serdang, Selangor, Malaysia; ‡ Australian Centre for Plant Functional Genomics, School of Agriculture and Food Science, The University of Queensland, St Lucia, Brisbane QLD 4072, Australia; § Centre of Excellence in Genomics (CEG), International Crops Research Institute for the Semi-Arid Tropics (ICRISAT), Patancheru 502324, Telangana, India; ** School of Plant Biology, University of Western Australia, Crawley, WA 6009, Australia

**Keywords:** abscisic acid, *Lotus japonicus*, mutagenesis, second-generation sequencing, SNP

## Abstract

Genetic structure can be altered by chemical mutagenesis, which is a common method applied in molecular biology and genetics. Second-generation sequencing provides a platform to reveal base alterations occurring in the whole genome due to mutagenesis. A model legume, *Lotus japonicus* ecotype *Miyakojima*, was chemically mutated with alkylating ethyl methanesulfonate (EMS) for the scanning of DNA lesions throughout the genome. Using second-generation sequencing, two individually mutated third-generation progeny (M3, named AM and AS) were sequenced and analyzed to identify single nucleotide polymorphisms and reveal the effects of EMS on nucleotide sequences in these mutant genomes. Single-nucleotide polymorphisms were found in every 208 kb (AS) and 202 kb (AM) with a bias mutation of G/C-to-A/T changes at low percentage. Most mutations were intergenic. The mutation spectrum of the genomes was comparable in their individual chromosomes; however, each mutated genome has unique alterations, which are useful to identify causal mutations for their phenotypic changes. The data obtained demonstrate that whole genomic sequencing is applicable as a high-throughput tool to investigate genomic changes due to mutagenesis. The identification of these single-point mutations will facilitate the identification of phenotypically causative mutations in EMS-mutated germplasm.

Mutagenesis provides a powerful technique to improve plant breeding and assist functional and genomic analyses of crop plants. This technique was first introduced with the use of x-ray and radium radiations followed by fast neutron and gamma radiation (as reviewed in Sikora *et al.* 2011). Because such application of physical mutagens required specialized equipment, chemical mutagens were introduced later. Chemical mutagens are used widely because they are easier to handle and increase mutation frequency (Loveless 1958; Sikora *et al.* 2011; Serrat *et al.* 2014). Various chemical mutagens have been prepared, such as sodium azide, ethyl methanesulfonate (EMS), and *N*-ethyl-*N*-nitrosourea, which produce different side effects on the genetic structure of treated populations. These chemicals can cause point mutations, insertions, and/or deletions in the genomic strands, leading to phenotypic changes, which could be desirable traits for important crops (Olsen *et al.* 1993; Greene *et al.* 2003; Flibotte *et al.* 2010).

EMS, an alkylating agent, commonly is used as a chemical mutagen for DNA lesions. Unlike *N*-ethyl-*N*-nitrosourea, EMS induces a biased spectrum of G/C-to-A/T transitions. These transitions most likely occur due to the alkylation at the O^6^ or N^7^ position of guanine, which leads to the replacement of cytosine with thymine base pairing (Lawley and Martin 1975; Sega 1984; Haughn and Somerville 1987; Sikora *et al.* 2011). Known as EMS canonical base substitutions, the high frequency of G/C-to-A/T changes has been observed upon EMS exposure in different organisms, including *Arabidopsis thaliana* (Greene *et al.* 2003; Till *et al.* 2011), *Oryza sativa* (Till *et al.* 2011), *L. japonicus* (Perry *et al.* 2009), *Caenorhabditis elegans* (Flibotte *et al.* 2010; Thompson *et al.* 2013), *Solanum lycopersicum* (Minoia *et al.* 2010), and *Saccharomyces cerevisiae* (Shiwa *et al.* 2012) at different rates. EMS also tends to produce random point mutations and induces a low level of chromosomal breaks and lethal effects (Greene *et al.* 2003). These effects provide a competent survival rate and allow subsequent analyses to be performed for both forward and reverse genetics.

The whole-genome sequence of *Lotus japonicus* ecotype *Miyakojima* (MG-20) is available, covering a total length of 315,073,275 bp (67% of the 472-Mb genome). A total of 91.3% of gene space is located in the genome sequence (Sato *et al.* 2008). The genome sequences of the chloroplast (150,519 bp) and mitochondrion (380,861 bp) also have been assembled, by Kato *et al.* (2000) and Kazakoff *et al.* (2012), respectively. Numerous bioinformatics materials on legume and nonlegume plants are also publicly available from various resources (Sato and Tabata 2005; Goodstein *et al.* 2012; Li *et al.* 2012). With the current high-throughput tools, genome sequencing can be performed at an affordable cost (Thudi *et al.* 2012). Many programs also have been developed for analyzing sequencing data *in silico*, offering good strategies to employ our study.

In this study, we applied Illumina second-generation sequencing (2GS) to discover EMS effects and the mutation spectrum in the genome of model legume, *L. japonicus* ecotype MG-20. We randomly selected two mutagenized plant genotypes from an M3 population as our subjects that were deep-sequenced. Wild-type (WT) MG-20 also was resequenced and mapped to the reference genome from Sato *et al.* (2008) as a comparison to subtract natural variations and false positives. We aimed to scan the effects of EMS throughout the whole genome, regardless of the phenotypic characteristic, which resulted from the mutations in specific regions. We identified single-nucleotide polymorphisms (SNPs) and compared the base alterations that occurred between both the genomes. The data demonstrate how 2GS works as a high-throughput platform for rapidly identifying DNA changes in each EMS-induced genome. As an advantage over sequencing pooled mutants, scanning individual mutagenized genomes allows rapid analysis of the mutation spectrum and gives the actual picture of “corrupted” genetic structure. The output of this study also will provide information in the identification of genes mutated due to EMS mutagenesis and demonstrate the distribution of mutation is comparable in different germplasm of MG-20.

## Materials and Methods

### Plant materials

A total of 4920 seeds of MG-20 were treated with 0.5% (v/v) EMS and grown as described in Biswas *et al.* (2009). One hundred MG-20 seeds were soaked in sterile water as a germination control. After 3 wk, the phenotypes of the plants that survived (67.3%) were observed to examine physical effects on the plant growth. Physiological tests also were performed as reported by Biswas *et al.* (2009) on abscisic acid (ABA) insensitivity and by Chan *et al.* (2013) on ethylene insensitivity. In this study, two mutated germplasms (M3) were isolated from the ABA assay (called AM and AS) for sequencing to screen EMS effects on their genome sequences and compare them with the resequenced WT MG-20 genome. In short, AM is a homozygous dominant, ABA-insensitive mutant (confirmed by stability in segregating families), whereas AS is the WT ABA phenotype segregant that was generated from a self-regeneration of a heterozygous ABA-insensitive mutant. Thus, AM and AS should carry the same spectrum of SNPs due to EMS mutagenesis.

### DNA extraction

Genomic DNA was extracted from each individual plant after 1 mo of growth. The cetyl trimethylammonium bromide (CTAB) extraction method was adapted from Stewart and Via (1993) with minor modification. Plant tissues (about 1 g) were ground to powder in liquid nitrogen before adding 1 mL of CTAB extraction buffer. The mixture was incubated at 65° for 30−60 min. Five-hundred microliters of the mixture was transferred into a new 2-mL tube and 500 µL of CTAB buffer was added to each tube. Both tubes were incubated as before. Chloroform purification was performed followed by isopropanol precipitation and washing with 70% ethanol. The nucleic acid containing pellet was air dried and dissolved in 100 µL of sterile water and subjected to RNase treatment (Sambrook and Russel 2001). Extracted genomic DNA was assessed using a spectrophotometer and agarose gel electrophoresis.

### Sequencing and SNP identification

Whole-genome paired-end, 100-bp, short-sequence reads (>10× coverage) for AM, AS, and WT MG-20 were generated using the Illumina Genome Analyzer IIx according to the manufacturer’s instructions. These three datasets were then mapped to the MG-20 reference genome (www.kazusa.or.jp/lotus/) using SOAP2 v2.21 with the option –r0 to retain only uniquely mapping read pairs (Li *et al.* 2009). SNPs were called using SGSautoSNP 2.001 (Lorenc *et al.* 2012) wherein AM, AS, and WT were referred as different cultivars. To avoid false-positive output, only homozygous SNPs were selected for further analysis. The three datasets of paired reads have been deposited into the National Center for Biotechnology Information Short Read Archive database under accession: SRX719550 (AM), SRX729747 (AS), and SRX131060 (WT). All custom scripts are available on request by E-mail at dave.edwards@uq.edu.au.

### SNP analysis

The resequenced WT genome was used for comparison to identify variants or base changes in the AS and AM genome sequences. The frequency of transitions and transversions generated in both mutated germplasms was also calculated. SNPs were categorized using SnpEff 3.0j (Cingolani *et al.* 2012) according to their effect on *L. japonicus* MG-20−annotated genes (Sato *et al.* 2008).

### Selection of genes involved in ABA perception and signaling pathways

A number of ABA candidate genes also were selected to test whether a SNP is located in their sequences. As a preliminary study, a total of 32 genes reported to be involved in ABA signaling were selected as candidate genes. These genes commonly are reported in the ABA-gene interaction in ABA perception and signaling pathways (Wasilewska *et al.* 2008; Cutler *et al.* 2010; Kim *et al.* 2010).

## Results

### Phenotypic effects

All control seeds successfully germinated and were well-developed. EMS-treated seeds had a germination rate of 67.3% (3313/4920). Among well-developed EMS-treated plants, 76 M1 plants showed abnormal phenotypes after 3 wk of growth. Almost 1% of these plants (30/3313) showed an albino phenotype, in which yellow sectors were observed. Some plants also had pale green patches (0.6%, 23/3313), early branching (0.2%, 7/3313) or a looped base (0.39%, 13/3313). Two other plants showed either unusual leaf shape or early flowering. Vivipary also was detected on a pod among this population. In this study, the impaired phenotypes of *L. japonicus* indicated the successful treatment of mutagenesis using 0.5% EMS, and therefore, the population could be utilized for subsequent analyses. We chose mutants at the third generation (M3) as subjects to ensure the mutation is stable and fixed (Serrat *et al.* 2014).

### Sequencing and read mapping output

A total of 32,965,291, 34,020,296, and 25,737,274 paired reads were generated from the WT, AS, and AM genomes, respectively (Table 1). At least 23% of reads from the sequenced genomes mapped to the reference. The percentage of mapped read pairs was 28.17%, 30.28%, and 23.59% from WT, AS, and AM, respectively, which resulted in more than 19× genome coverage. As a result, the genome coverage was sufficient to be applied for SNP calling between mutants and WT reads.

**Table 1:** Outputs generated from Illumina sequencing to read mapping

Genome	Paired Raw Reads	Read Pairs Mapped	% of Read Pairs Mapped	Genome Coverage*^a^*
MG-20 WT	32,965,291	9,285,440	28.17	29.88X
AS mutant	34,020,296	10,299,840	30.28	33.15X
AM mutant	25,737,274	6,071,230	23.59	19.54X

WT, wild type.

a Based on mapped reads.

### Frequency of mutation

After read mapping, homozygous SNPs were predicted, to identify SNPs that are unique in the mutant genome as opposed to WT. As a result, the frequency of mutation could be observed in assembled sequences of all chromosomes (Table 2). Chromosomes 1 and 6 have the longest and shortest length of assembled sequences, respectively. Meanwhile, the unmapped contigs cover approximately 32.9 Mb of total assembled length. Our SNP calling showed that Chromosome 1 has the greatest number of SNPs with one mutation per 170 kb and 165 kb in the AS and AM genomes, respectively. Mutation frequency was the lowest in Chromosome 6, which had less than 6% of total SNP in both genomes. The change rates were one mutation per 222 kb for AS and 217 kb for AM. In total, mutation frequency in AM and AS was nearly identical 1490 SNPs and 1447 respectively. This frequency is reflected in the change rate of mutation in the AM genome (one homozygous mutation in every 202 kb) and AS genome (one homozygous mutation in every 208 kb).

**Table 2:** Frequency of SNPs in individual chromosomes and unmapped regions of AS and AM mutants

		AS Genome		AM Genome	
Chromosome	Length, bp	Change (SNPs)	Change Rate	Change (SNPs)	Change Rate
1	66,776,104	391	170,783	404	165,287
2	44,510,304	258	172,521	250	178,041
3	48,258,781	208	232,013	204	236,563
4	43,347,107	176	246,290	195	222,293
5	37,320,184	190	196,422	200	186,601
6	28,216,978	76	371,276	86	328,104
Unmapped	32,912,371	148	222,381	151	217,963
Total	301,341,829	1447	208,253	1490	202,243

Total of assembled length (bp), base changes, and change rate of individual chromosomes and unmapped regions are listed in the table. SNPs, single-nucleotide polymorphisms.

The frequency of transitions and transversions generated was analyzed (Table 3). In our mutants, EMS has generated 64.7% (AS) and 62.3% (AM) transitions as a percent of total mutation. Both mutants showed a bias to G/C-to-A/T transitions, which were the most frequent mutations that occurred (45.0% in AS and 34.9% in AM). Transversion mutation was also detected, but at lower percentages as listed in Table 3. The lowest percentage of mutation was C/G-to-G/C changes in AS and A/T-to- T/A changes in AM.

**Table 3:** Spectrum of base mutation found in the AS and AM genomes

	Changes, %	
Mutation	AS	AM
Transition		
G/C-to-A/T	45.0	34.9
** **A/T-to-G/C	19.7	27.4
Transversion		
A/C-to-C/A	12.7	15.3
G/T-to-T/G	12.6	13.3
A/T-to- T/A	6.9	4.5
C/G-to-G/C	3.1	4.6

A high frequency of transition mutation was observed as expected

### Distribution of transition and transversion mutations

**Figure 1:**
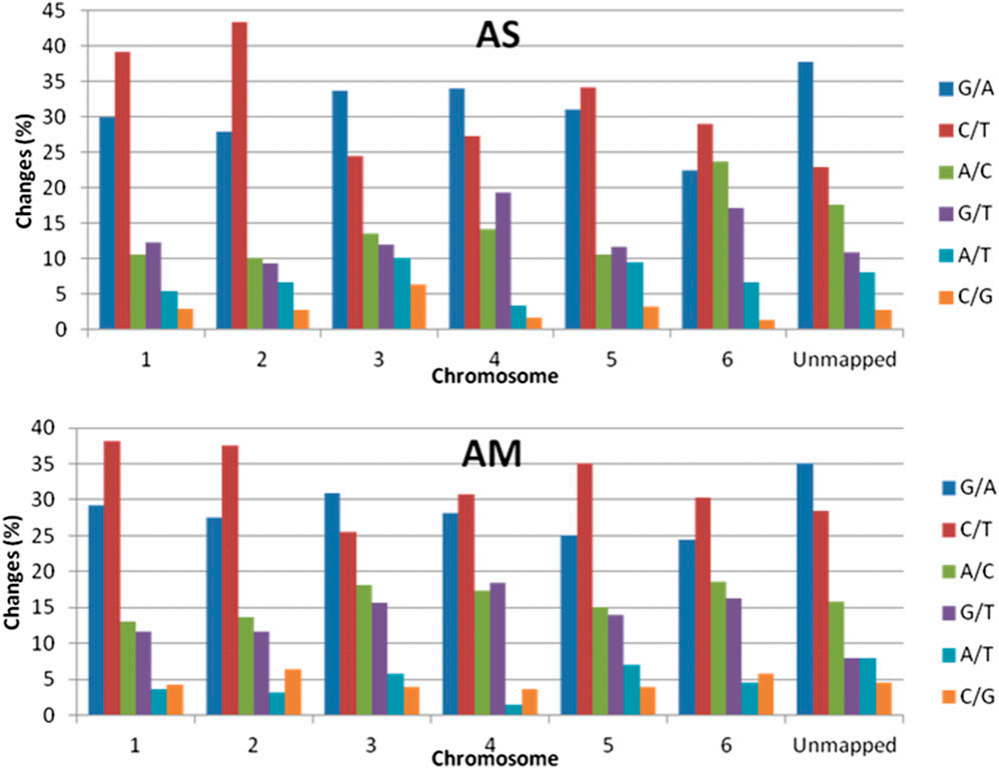
Relative percentage of different mutation types. Our mutated genomes had varied percentages of base changes in each chromosome and unmapped regions. A high percentage of G/A and C/T changes was observed in both genomes.

Based on the mutation frequency, how each mutation type was located in individual chromosomes can be observed by calculating the percentage of mutation type in relation to the SNP total in each chromosome (Figure 1). Both genomes comprised of C/T or G/A transitions as the most frequent mutation type in their chromosomes or unmapped regions. In the AS genome, G/A transitions were the highest in Chromosomes 1, 2, 5, and 6. Chromosomes 3 and 4 and unmapped regions had C/T transitions as the highest percentage of mutation type. The lowest percentage of mutation type was C/G transversions which were present the least in all individual chromosomes and unmapped regions of the AS mutant. The distribution of transitions and transversions in the AM genome was relatively similar to the AS genome. The greatest percentage of mutation type in each chromosome of the AM genome was C/T transitions except Chromosome 3. Unmapped regions and Chromosome 3 of AM have G/A transitions as the greatest percentage of mutation type. Meanwhile, the lowest percentage of mutation type was detected to be either A/T or C/G transversions in the AM genome.

### Distribution of SNPs across mutagenized genomes

**Figure 2:**
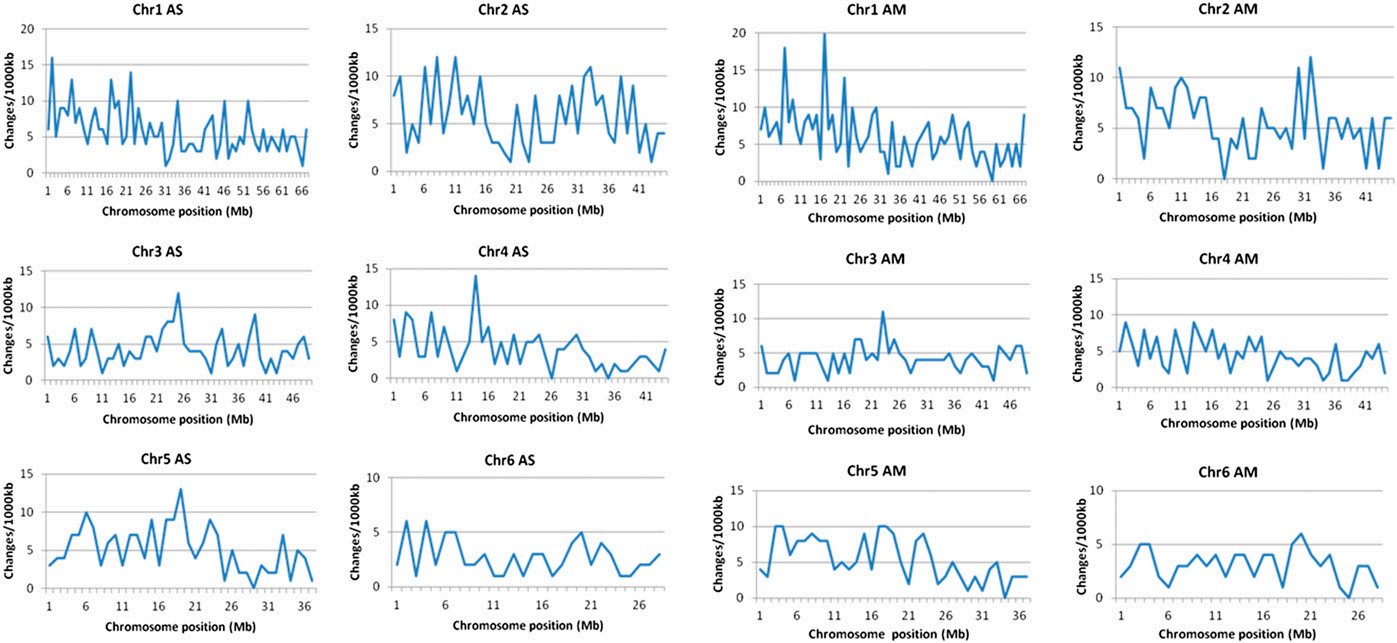
Distribution of mutation across individual chromosomes in both AS (left) and AM (right) genomes. Mutations were plotted in every 1000 kb of genomic sequences. Chromosome (Chr) number is shown above its designated graph. Chromosome position was plotted based on the assembled length of *L. japonicus* genome.

We determined how many SNPs occurred every 1000 kb to show the distribution of mutations across our mutagenized genomes (Figure 2). Regardless of mutation types, the distribution of SNPs is unique between AS and AM when comparing the same chromosome. SNPs were randomly located along the genomes with no specific chromosome position being particularly abundant or lacking in SNPs for both genomes. However, Chromosomes 1 and 2 were highly “corrupted” in their arms. Meanwhile, some chromosomes (Chromosomes 3, 4, and 5 for AS; Chromosome 3 for AM) have a high peak of SNPs toward their center. The density of mutations in every 1000 kb was relatively apparent in Chromosomes 1 and 2 of AS and AM. Meanwhile, Chromosome 6 had less dense mutations for both genomes.

### Coding and noncoding effects

**Figure 3:**
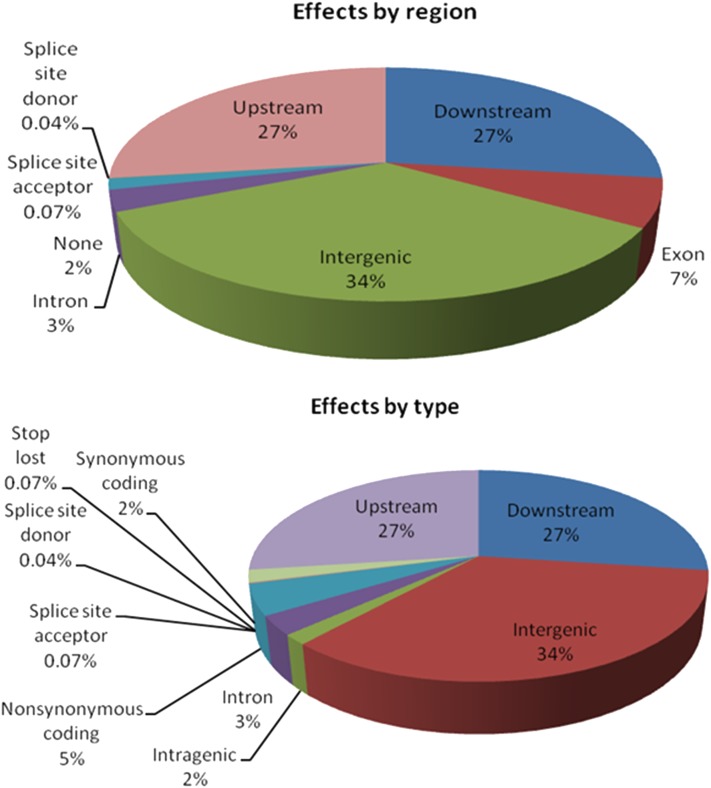
Mutation effects on codon sequences by type and region in our mutant genomes. Single-nucleotide polymorphisms were observed highly located in intergenic regions, upstream, and downstream parts of the annotated genes. Only a small percentage of nonsynonymous changes was predicted.

Here, we used SnpEff to predict the effect of mutations on coding regions of annotated genes of *L. japonicus* for both mutants, as shown in Figure 3. Our results showed that the greatest number of SNPs (34%) was predicted to be located in intergenic regions, followed by downstream and upstream regions of predicted genes (27% each). Only 7% and 3% of SNPs were located in exon and intron sequences, respectively. Intragenic regions also were predicted to have low EMS effect. The mutation percentage was very low at splice site donor and acceptors. In our mutagenized genomes, EMS effects did not contribute to a high fraction of nonsynonymous and synonymous changes (5% and 2%, respectively).

### Candidate genes

As preliminary research, we selected 32 notorious ABA genes (Table 4) to determine whether any SNPs were located in their sequences. Orthologous genes of *L. japonicus* were identified based on *A. thaliana* and *Glycine max* sequences. Six were found to have orthologous sequences, which were positioned in unmapped regions of the MG-20 genome. The rest of the orthologs were found in the assembled chromosomes. SNPs were identified in three candidate loci, which were ABI2, ABI3, and ABI4. ABI2 and ABI4 were mutated in both mutants, whereas SNP was only found in ABI3 of the AS mutant.

**Table 4:** List of ABA candidate genes and their loci in the genome of *Arabidopsis*, soybean, and *Lotus*

No.	Candidate Gene	Molecular Function	Locus		
			*Arabidopsis thaliana*	*Glycine max*	*Lotus japonicus*
1	*ABA INSENSITIVE 2* (*ABI2*)	Protein phosphatase 2C	AT5G57050	Gm17g33410.1	chr1.CM0133.740.r2.m
2	*ABI3*	Transcription factor	AT3G24650	Gm08g47240.1	chr1.CM0147.920.r2.d
3	*ABI4*	Transcription factor	AT2G40220	Gm02g31350.1	chr1.CM0318.160.r2.d
4	*ABI5*	bZIP transcription factor	AT2G36270	Gm19g37910.1	chr1.CM0010.100.r2.d
5	*ABI8*	Glycosyl transferases	AT3G08550	Gm04g26230.1	chr3.CM2163.130.r2.m
6	*AGB1*	Heterotrimeric G-protein complex	AT4G34460.1	Gm11g12600.1	chr1.CM0113.1970.r2.d
7	*AHA1*/ *OPEN STOMATA 2* (*OST2*)	ATPase	At2G18960.1	Gm13g00840.1	chr4.CM0244.50.r2.m
8	*ABA Responsive Element-binding Factor2* (*ABF2*)/*AREB1*	bZIP transcription factor	AT1G45249.3	Gm06g04350.1	chr1.CM2113.380.r2.a
9	*ATPT2*/ *PHT1*:4	Phosphatase transporter	AT2G38940	Gm19g34710.1	chr1.CM0295.140.r2.m
10	*Ethylene Response DNA binding factor 3* (*EDF3*)	Transcription factor	AT3G25730	Gm10g34760.1	LjSGA_080421.2
11	*Ethylene Response Factor 7* (*ERF7*)	Transcription factor	At3G20310	Gm14g02360.1	LjSGA_013296.1.1
12	*Enhanced Response to ABA 1* (*ERA1*)	Farnesyltransferase activity	AT5G40280	Gm13g23780.1	chr2.CM0081.550.r2.d
13	FUS3	Transcription factor	AT3G26790.1	Gm19g27340.1	chr1.CM0104.400.r2.a
14	*Gated Outwardly Rectifying K+ Channel* (*GORK*)	Outward rectifier K channel activity	AT5G37500.1	Gm02g41040.1	chr6.CM0508.670.r2.m
15	*Stelar K+ Outward Rectifier* (*SKOR*)	Outward rectifier K channel activity	AT3G02850	Gm14g39330.1	Same as GORK
16	*GPA1*	GTP binding protein	AT2G26300.1	Gm06g05960.1	chr5.CM0034.250.r2.m
17	*Glutathione peroxidase 3* (*GPX3*)	Redox transducer & scavenger	AT2G43350.1	Gm11g02630.1	chr4.CM0004.300.r2.m
18	*GPCR-TYPE G protein 1* (*GTG1*)	ABA receptor	AT1G64990.1	Gm12g0174	chr3.CM0127.40.r2.m
19	*High Leaf temperature 1* (*HT1*)	Serine/threonine protein kinase	AT1G62400	Gm07g39460.1	chr4.CM0288.800.r2.m
20	*Keep On Going* (*KEG*)	Ring E3 ligase	AT5G13530	Gm11g25680.1	LjSGA_007856.1
21	*Potassium Channel In A. thaliana 1* (*KAT1*)	Cyclic-nucleotide binding	AT5G46240	Gm08g24960.1	chr6.CM157.280.r2.a
22	*OST1*	Serine/threonine protein kinase	AT4G33950.1	Gm02g15330.1	LjSGA_038133.1
23	*PLDα1*	Phospholipase D/transphosphatidylase	AT3G15730.1	Gm07g03490.1	chr3.CM0142.570.r2.d
24	*Pyrabactin Resistance1-like 2* (*PYL2*)/*Regulatory Component of ABA Receptor 14* (*RCAR14*)	Polyketide cyclase/dehydrase	AT2G26040	Gm04g05380.1	LjSGA_056222.1
25	*PYL3*/*RCAR13*	Polyketide cyclase/dehydrase	Similar to PYL2	Similar to PYL2	Similar to PYL2
26	*PYL4*/*RCAR10*	Polyketide cyclase/dehydrase	AT2G38310	Gm18g37410.1	chr3.CM0116.270.r2.m
27	*PYL5*/*RCAR8*	Polyketide cyclase/dehydrase	AT5G05440	Gm02g42990.1	LjSGA_020312.1
28	*Pyrabactin Resistance1* (*PYR1*)/*Regulatory Component of ABA Receptor 11* (*RCAR11*)	*Streptomyces* cyclase/dehydrase	AT4G17870.1	Gm08g36770.1	chr2.CM0177.730.r2.m
29	*Regulator of G protein Signaling 1* (*RGS1*)	G-protein coupled receptor	AT3G26090	Gm11g37540.1	chr6.LjT45M05.110.r2.d
30	*TPK10*/*CIPK15*	CBL-interacting serine/threonine protein kinase	AT5G01810.1	Gm18g44450.1	chr3.LjT45I18.90.r2.d
31	*Slow Anion Channel Associated 1* (*SLAC1*)	C4-dicarboxylate transporter/malic acid transport	AT1G12480	Gm09g23220.1	LjSGA_063103.1
32	*MYB44*	Transcription factor	None	Gm04g04490.1	chr5.CM0096.100r2.m

ABA, abscisic acid.

## Discussion

The effect of EMS mutagenesis initially can be observed through phenotypic changes of mutagenized plants, and numerous reports have been published on the effects. The abnormal phenotypic variance due to EMS exposure also has been reported previously in *L. japonicus* (Perry *et al.* 2003) and other plants such as *Glycine max* (soybean; Carroll *et al.* 1986), *Hordeum vulgare* L. (barley; Caldwell *et al.* 2004), *Sorghum bicolor* (L.) Moench Xin *et al.* 2008), *Solanum lycopersicum* (tomato; Minoia *et al.* 2010), and *Capsicum annuum* L. (chilli pepper; Sri Devi and Mullainathan 2012). The percentage of the abnormal phenotypes varied in different plants depending on the concentration of EMS used (Xin *et al.* 2008). Phenotypic changes are not restricted to plant growth but also affect their physiological characteristics. For examples, reduced sensitivity to ABA was reported by Biswas *et al.* (2009) in *L. japonicus* mutagenized by EMS. Response to high temperature also was affected in EMS-induced *Arabidopsis* mutants impaired in ABA and salicylic acid syntheses (Zhu *et al.* 2012).

Sequencing data often are used to mine SNPs or polymorphisms among different cultivars for identification of traits and allelic variations in crops (Varshney *et al.* 2012; Edwards *et al.* 2013; Zander *et al.* 2013). The high-throughput technology also is applied in discovering causative mutations by pooling backcrossed segregant populations to increase SNP frequency and reveal mutated regions (Ashelford *et al.* 2011; Mokry *et al.* 2011; Hartwig *et al.* 2012). Here, we individually sequenced two selected mutant genomes and used 2GS data to compare mutation distribution between these mutants.

To our knowledge, this is the first report on the application of SGSautoSNP for discovery of SNPs induced by EMS mutagenesis. This tool uses assembled reads to identify homozygous SNPs without using the reference genome, which is only used for mapping reads. SGSAutoSNP was used to identify only homozygous SNPs because the identification of heterozygote SNPs leads to a high number of false positives, which would lead to an overrepresentation of the mutation frequency in the genome (Lorenc *et al.* 2012). We also managed to rapidly identify the mutation spectrum occurring in the mutant genomes using the output data.

Our read mapping has been successfully performed before SNP identification in each genome. However, a low percent of reads was mapped to our reference. A high percentage of paired reads was reported to map to the reference in other species such as *Caenorhabditis elegans* (average 92%; Zuryn *et al.* 2010) and *A. thaliana* (average 73%; Austin *et al.* 2011). In our case, only an average of 27% paired reads mapped. Similar results were observed in other species using the same SNP prediction software [for example in canola (Dalton-Morgan *et al.* 2014) and wheat (Lai *et al.* 2015)]. The number of reads mapped relies on the parameters set up during the mapping procedure. We only selected paired reads that mapped and ignored reads that aligned as single reads and only used reads that mapped to a single unique location to generate more accurate read mapping (Li *et al.* 2009; Lorenc *et al.* 2012). The assembled length of pseudomolecules in our MG-20 reference covers 67% of estimated genome size (Sato *et al.* 2008), which also influences read mapping output. In addition, reads that aligned to multiple positions also were removed to increase SNP calling accuracy and avoid false positives (Lorenc *et al.* 2012; Shiwa *et al.* 2012). These factors have reduced the number of reads mapped in this study.

Chromosome 3 is the largest among the six chromosomes of the MG-20 genome. However, the assembled length of Chromosome 1 is the longest, and presumably more complete, based on the genome assembly (Sato *et al.* 2008), allowing for better read mapping and a greater rate of SNP identification. This reflects the greatest number of SNPs predicted in Chromosome 1. Chromosome 6 has the lowest number of identified SNPs, because it is the shortest chromosome in assembled length (Sato *et al.* 2008). In addition, Chromosome 6 might contain more repetitive sequences and therefore less unique reads mapped, leading to a lower rate of SNPs being identified. Furthermore, the different rates of base changes between chromosomes may demonstrate the different capacity levels of each chromosome in tolerating mutation impact. There is insufficient evidence to reach clear conclusions; however, the presence of selection pressure, such as clustering of housekeeping genes on chromosomes, could contribute to inability to tolerate mutations. Additionally, not all SNPs will be identified across the genome due to repetitive regions and the stringency of read mapping, reflecting SNP/mutation density across chromosomes.

A different number of SNPs between AS and AM was expected, because it is impossible to obtain an exact total of SNPs in different mutated genomes. The mutation rate of our mutants was 1/208 kb and 1/202 kb, which were greater than previously reported in *L. japonicus* (1/502 kb; Perry *et al.* 2009). Different rates of mutation also were reported in various plants mutagenized by EMS, as summarized by Sikora *et al.* (2011). Although only homozygous SNPs were taken into account in this study, the frequency of mutation was quite high. If heterozygous mutations were called, the frequency will be greater. Here, the frequency of mutation demonstrates the effectiveness of EMS to produce high mutagenesis as reported by many researchers (Dube *et al.* 2011; Shiwa *et al.* 2012; Serrat *et al.* 2014).

The mutation spectrums in our germplasms were not consistent with a previous report on TILLING work of mutagenized *L. japonicus Gifu* (Perry *et al.* 2009) wherein 97.6% of base changes were G/C-to-A/T mutation upon EMS mutagenesis. Similar to TILLed *Arabidopsis*, a high frequency of G/C-to-A/T transition (99%) also was identified (Greene *et al.* 2003). A lower rate of G/C-to-A/T base changes was observed in other plants, including barley (70%; Caldwell *et al.* 2004), rice (70%; Till *et al.* 2007), and tomato (60%; Minoia *et al.* 2010), showing a possibility of the presence of various base changes upon EMS exposure. However, transition mutations could not be denied as the most common base mutation in the EMS sphere as reported previously (Lawley and Martin 1975; Sikora *et al.* 2011; Shiwa *et al.* 2012). In the TLLING analysis, a number of interesting genes with known sequences were selected for the identification of base changes in pooled mutated genomes (McCallum *et al.* 2000). In this study, we adopted a high-throughput technology (2GS) to scan homozygous SNPs that are present throughout the individual mutated genomes compared with our resequenced WT MG-20. This provided a wider range of EMS effects in a genome. Our approach also neglected natural variation between the reference and the resequenced MG-20 genomes. Our reference was used merely for read mapping and has not been counted during SNP calling (Lorenc *et al.* 2012).

Although the percentage of transversion mutations was relatively low in both genomes, they should not be omitted in a mutation study because they potentially are causal mutations for phenotypes of interest. Taking into account only homozygous mutations, our data also show that the presence of specific base changes is comparatively distributed among individual chromosomes and not abundantly located in a specific chromosome for both alleles. Comparing these two genomes, we found that transition and transversion mutations were present nearly consistently in the same chromosomes. These results reveal that the frequency of EMS-induced transition and transversion mutations is comparable between different individual mutants that were derived from the same mutagenized population.

As mentioned previously, the total assembled length affects the SNP total identified in each chromosome and consequently, their distributions. The longer the assembled length, the denser the mutation distribution was found to be along the chromosome. The mutation distribution also was affected by the occurrence of assembled sequences at specific regions (www.kazusa.or.jp/lotus/). Chromosome 6 is the best example, in which low SNP peaks were detected and the mutations distributed broadly. Read mapping is very difficult in centromeric regions, and therefore the density in these regions has not been investigated in this study. Distribution of mutations was scattered due to the effect of EMS as a mutagen that causes a random mutation (Lawley and Martin 1975; Greene *et al.* 2003; Sikora *et al.* 2011; Tagu *et al.* 2014). A random distribution also was reported by Thompson *et al.* (2013) in the genome of single *C. elegans* from different strains, which were mutagenized by EMS. They propagated mutagenized worms through 10 generations to obtain stable mutants. However, this is time-consuming for legumes, which have a longer generation period. Nevertheless, our 2GS data of third-generation mutants also could provide the literal effect of EMS mutagenesis throughout the individual genomes.

EMS induces base changes or nucleotide substitution, which consequently alter codon sequences, leading to either nonsynonymous or synonymous effects. In genetics studies, nonsynonymous change is a favorable mutation effect because it gives a clue on which gene may be associated with a specific phenotype (Ng and Henikoff 2006). Our results showed that EMS has arbitrary and broad effects of mutation on codon sequences. Mutated coding regions could represent nonsynonymous changes, which are useful to identify gene of interest for desirable phenotypes.

To extend the implication of our data, preliminary work has been employed to specifically detect the presence of mutations in our genes of interest. A total of 32 genes reported to be involved in ABA signaling were selected as candidate genes. These genes are commonly reported in the ABA−gene interaction in ABA perception and signaling pathways (Wasilewska *et al.* 2008; Cutler *et al.* 2010; Kim *et al.* 2010). We chose to use ABA candidate genes because our mutants were isolated from a mutagenized population effected in response to ABA. We did identify base changes in several candidate genes, indicating a low rate of EMS effects on these sequences. In addition, identified SNPs were not only in the ABA-insensitive AM mutant, indicating they were background mutations. On the other hand, comparing mutations between AM and AS mutants would provide clues on the causal mutation of mutant phenotypes. However, other candidate genes also can be considered because ABA is involved in a wide range of plant growth and responses to environmental stresses. Its effects are varied in regulating the events of plant physiology, which require gene interaction and/or cross talks with other hormones (as reviewed in Kermode 2005; Fujita *et al.* 2006; Rock *et al.* 2010). Because this research worked on an individual genome of mutants, another strategy has been developed to sequence pooled DNA from different mutants to remove the effect of background mutation and increase the likelihood of identifying the causative gene.

In conclusion, this scanning revealed a detailed effect of EMS mutation on the whole genome of an individual mutant. Our results presented an overview of point mutations that occurred in the genome of mutants, which were usually pooled to identify SNPs. Here, EMS has produced a number of abnormal plants in our mutant population of *L. japonicus*. Our 2GS data also revealed how EMS efficiently mutates genomic sequences in an individual mutagenized plant. As expected, EMS created a random spectrum of mutation across the whole genome of our mutants and biased to G/C-to-A/T changes. However, other transition and transversion mutations also were identified with quite apparent fractions. Calling only homozygous SNPs has put a high confidence in identified base changes occurred. Mutation distribution apparently is distinct between mutated genomes, which derived from the same mutagenised population. Effects of SNPs on coding and noncoding regions could be manipulated to identify a causal mutation of a phenotype of interest. Our next question will be which gene is responsible for our abnormal phenotype of interest. This 2GS data will be further analyzed to discover the causative mutated gene.
